# Development of an enterprise risk inventory for healthcare

**DOI:** 10.1186/s12913-018-3400-7

**Published:** 2018-07-24

**Authors:** Ana Paula Beck da Silva Etges, Veronique Grenon, Ming Lu, Ricardo Bertoglio Cardoso, Joana Siqueira de Souza, Francisco José Kliemann Neto, Elaine Aparecida Felix

**Affiliations:** 1 School of Technology, PUCRS, Avenida Ipiranga, 6681, Porto Alegre, 90619-900 Brazil; 2 National Health Technology Assessment Institute, CNPq, Porto Alegre, RS Brazil; 3 Department of Industrial Engineering, UFRGS, Porto Alegre, RS Brazil; 4 The Risk Authority Stanford, Palo Alto, California, USA; 5 Department of Anesthesiology, School of Medicine, UFRGS, Porto Alegre, RS Brazil; 6 Guy Carpenter, LLC, New York, NY USA

**Keywords:** Enterprise risk management, Healthcare management, Risk inventory, Healthcare, Risk identification, Risk analysis

## Abstract

**Background:**

The first phase of an enterprise risk management (ERM) program is the identification of risks. Accurate identification is essential to a proactive and effective ERM function. The authors identified a lack of such risk identification in the literature and in practical cases when interviewing the chief risk officers from healthcare organizations. A risk inventory specific to healthcare organizations that includes detailed risk scenarios and risk impacts currently does not exist. Thus, the objective of this research is to develop an enterprise risk inventory for healthcare organizations to create a common understanding of how each type of risk impacts a healthcare organization.

**Method:**

ERM guidelines and data from 15 interviews with chief risk officers were analyzed to create the risk inventory. The identified risks were confirmed through a survey of risk managers from a range of global healthcare organizations during the ASHRM conference in 2017. Descriptive statistics were developed and cluster analysis was performed using the survey results.

**Results:**

The risk inventory includes 28 risks and their specific risk scenarios. Cyberattack was ranked as the principal risk by the participants, followed by sentinel events and risks associated with human capital management (organizational culture, use of electronic medical records and physician wellness). The data analysis showed that the specific characteristics of the survey participants, such as the length of time working in risk management, the size of the organization, and the presence of a school of medicine, do not impact an individual’s opinion of the importance of the risks identified. A personal background in risk management (clinical or enterprise) was a characteristic that showed a small difference in the perceived importance of the risks from the proposed risk inventory.

**Conclusions:**

In addition to defining specific risk scenarios, the enterprise risk inventory presented in this research can contribute to guiding the risk identification phase of an ERM program and thereby support the development of a risk culture. Patient data security in hospitals that operate with high levels of technology is fundamental to delivering high quality and safe care to patients. At the top of the risk ranking, the identification of cyberattacks reflects the importance that healthcare risk managers place on this risk by allocating time and other resources. Exploring opportunities to improve cyber risk management and evaluating the benefits of using the risk inventory at the beginning of the risk identification phase in an ERM program are suggestions for future studies.

**Electronic supplementary material:**

The online version of this article (10.1186/s12913-018-3400-7) contains supplementary material, which is available to authorized users.

## Background

Enterprise risk management (ERM) programs have been implemented in organizations across various industries with the aim of minimizing the negative effects of uncertainty in achieving corporate objectives while at the same time promoting its potential positive effects [1, 2]. As stated in the most recent guidelines, ERM programs facilitate strategy selection. Choosing a strategy calls for a structured decision-making process that analyzes risks and aligns an organization’s resources with its mission and vision [3]. In the healthcare industry, the ERM process has been explored by risk managers to improve the organizational value creation process and develop a safer environment [4, 5].

ERM guidelines, including ISO 31000 [6, 7] and COSO [3, 8], outline an ERM process that includes several common phases: identification, analysis, assessment, monitoring and control. Adequately performing the first phase, risk identification, is a requirement to build a proactive and effective ERM process [9, 10]. In the same way that Cox’s (2008) [11] research explores how risk matrices can be used in the ERM process during the risk analysis phase, this research takes a deep dive in the risk identification phase. The ability to identify and define risks correctly is indispensable to subsequently enable the effective use of risk analysis tools [10, 12].

The risk identification process needs to be proactive, to involve multiple employees, and to create value for and protect the organization [13, 14]. In previous research that explored how ERM is conducted in healthcare organizations, it was established that the guidelines that currently exist are not practical because they only include a list of risk domains [12]. The development of an enterprise risk inventory that includes specific risk events, details of the risk scenarios and descriptions of how each risk impacts the organization was identified as a gap for healthcare organizations.

The guidelines by the Committee of Sponsoring Organizations of the Treadway Commission (COSO) were the first to define risk factors by industry, but they do not explore risk events in detail. In 2014, the American Society of Healthcare Risk Management (ASHRM) proposed risk domains for healthcare organizations, but again, risk events and scenarios are not described in detail [15]. Other institutions, such as Healthcare Insurance Reciprocal of Canada (HIROC) [16] and the National Health Service in England (NHS) [17] have developed risk taxonomies that include clinical risks and enterprise risks. In 2014, AON Corporation published the Healthcare Industry Report [18] based on collaborative research with various healthcare organizations that proposed ten common healthcare risks: regulatory/legislative changes; failure to attract or retain top talent; economic slowdown/slow recovery; increasing competition; damage to reputation/brand; failure to innovate/meet customer needs; lack of technology infrastructure to support business needs; political risk/uncertainties; workforce shortages; and cash flow/liquidity. Unfortunately, and similar to other existing guidelines, this report does not define each risk in sufficient detail for multiple individuals in an organization to have a common understanding of the risks the organization faces. This means that every healthcare organization must develop its own enterprise risk identification process.

The authors previously interviewed 15 hospital risk officers from Brazil and the USA and presented a novel model for healthcare risk management, the Economic Enterprise Risk Management innovation program for healthcare: E^2^RM_healthcare_ [19]. This previous research identified qualitative differences in individual risk perception capabilities among risk managers from large and small hospitals based on personal background and whether the hospitals were associated with a school of medicine. To complement the published model, the authors reviewed the data again and conducted a new survey in order to develop an enterprise risk inventory for use at the beginning of the risk identification phase.

Thus, the main objective of this paper is to develop an enterprise risk inventory for healthcare organizations in order to create a common understanding of how each type of risk impacts a healthcare organization. Additionally, it aims to determine whether the length of time working with ERM, the number of employees at the hospital and the presence of a school of medicine impact the perceived importance of the enterprise risks identified.

## Methods

**Fig. 1:**
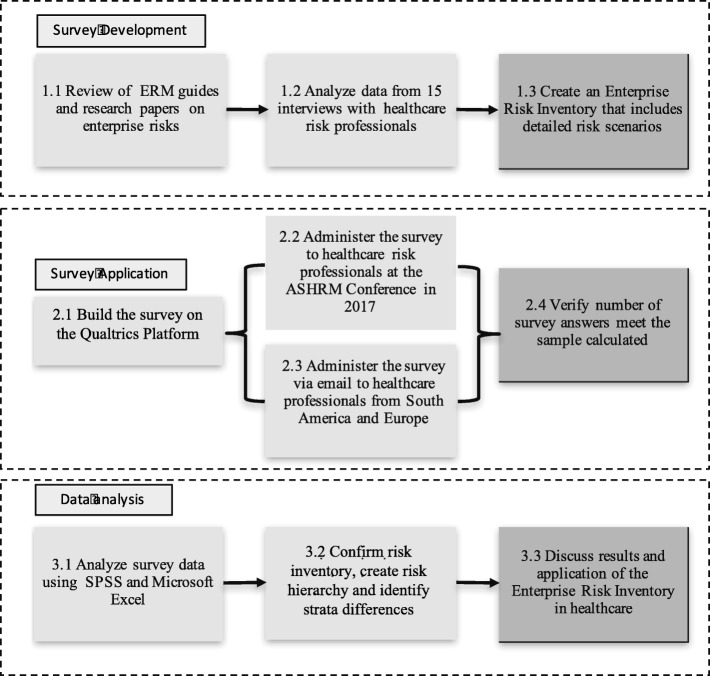
Research methods

This study can be classified as exploratory, as it analyzes the literature and data collected from interviews to increase the knowledge about ERM [20]. Thus, a survey was constructed and administered, data from the survey responses were collected, and a quantitative analysis was performed. Figure 1 illustrates the three phases: survey development, survey application and data analysis.

### Survey development

To construct the survey, two steps were taken. First, data from 15 interviews with risk professionals from various healthcare organizations in Brazil (7) and the United States (8) were analyzed. Hospitals in Brazil were identified using a list from the magazine *America Economia* (2014) as “the best hospitals in Latin America”. JCI-accredited hospitals and hospitals with risk management teams in their management structure were selected and contacted. US hospitals with national quality accreditations as well as established risk management teams were also contacted. Data from a ninth US hospital, however, were not included due to incompleteness, which prevented comparisons. The resulting sample was heterogeneous, as it included data from different types of organizations: private and public hospitals, academic and non-academic hospitals, and a range of sizes. The main characteristics of the healthcare organizations interviewed are presented in Additional file 1. Second, the content of the guidelines developed by COSO (2007) [8], ASHRM (2014) [15], HIROC (2014) [16], NHS (2008) [17] and AON (2014) [18] were assessed, as they were mentioned by the interviewees as being important to the creation of their ERM programs.

The software NVIVO was used to analyze the combined content of the interviews and guidelines. The researchers used the software to identify the risks listed by the interviewees to develop a first enterprise risk inventory list based on repetition of the risks by the interviewees and the literature. In sequence, two external risk management consultants, one Brazilian and the other from USA, both of whom had more than 10 years of experience in healthcare risk management, discussed the risk inventory with the two first authors of this study. The inventory was agreed upon by the authors, including the name of the risk, the concept that it described, and a detailed risk scenario. The risk scenarios considered real examples that occurred in recent years in hospitals throughout the world that were shared on global media.

Subsequently, the survey was built using the Qualtrics platform. The survey was made available online, and the participants were asked to choose if they strongly agree, somewhat agree, neither disagree nor agree, somewhat disagree or strongly disagree when asked about the importance of each risk identified. The complete questionnaire can be found in Additional file 2 through an online link.

### Survey application

A stratified approach was used to calculate the minimum number of surveys that needed to be completed. Two variables for stratification were defined: length of time working in risk management and type of risk management (clinical or enterprise). These variables were selected based on the results presented by Etges et al. in previous research [19]. The 15 interviews were analyzed to develop an ERM model oriented toward healthcare organizations. The model also presented the differences between clinical and enterprise managers and those related to length of working time in risk management. For each stratum variable, two classes were identified: (stratum 1) number of years working in risk management – less than 7 years and more than 7 years; and (stratum 2) type – clinical risk management and enterprise risk management. The total number of strata is therefore four. To calculate the minimum number of questionnaires per group, a normal distribution was used. The formula to calculate the number of questionnaires per group is defined in eq. 1:

1 $$ n={Z}_{\frac{\alpha }{2}}^2\kern0.5em \frac{CV^2}{ER^2} $$

${Z}_{\frac{\alpha }{2}}^2$ = significance level to be applied in the estimation;

*CV*^*2*^ = coefficient of variation;

*ER*^*2*^ = the permissible relative error, that is, the percentage error in the estimate that we were willing to accept.

Assuming a significance level of 5%, ${Z}_{\frac{\alpha }{2}}^2=1.96$, with a moderate CV and a low ER, we calculated 16 completed surveys per group and a total of 64 completed surveys for the four groups combined.

In October 2017, the American Society of Healthcare Risk Management’s annual conference took place in Seattle. The survey was distributed at the conference during ERM workshops and at the exhibit hall, where only people participating in the conference had access. In parallel, emails were sent to various healthcare risk professionals in Brazil and United States who worked at tertiary hospitals and occupied a risk management position. The survey was open from October 10, 2017 to January 5, 2018.

### Data analysis

The survey data were extracted from Qualtrics and analyzed using SPSS and Microsoft Excel software. The descriptive statistical analysis was used to create a risk ranking and analyze differences between the strata. The risk ranking was first analyzed based on the Likert scale.

The second, third and fourth analyses utilized a binary reference. The answers “strongly agree” and “somewhat agree” were classified as agreeing that the risk is an important enterprise risk in the healthcare industry. The answers “strongly disagree”, “somewhat disagree” and “neither agree nor disagree” were classified as not agreeing that the risk is an important enterprise risk in the healthcare industry. The second analysis combined different sample strata (time working in risk management and type of risk management background, clinical or enterprise). The third analysis compared the survey results between participants who worked in organizations with more than 1000 employees to those who worked in organizations with fewer than 1000 employees. The fourth analysis compared the participants’ opinions from organizations with and without a school of medicine.

Cluster analysis was performed to allocate the risk professionals to groups based on their answers regarding the perceived importance of each risk. The cluster classification was performed in the software SPSS in two steps following Favero et al. (2009) [21]. First, the hierarchical algorithm nearest neighbor was applied to the data, which enabled the number of clusters to be defined through an analysis of the resulting dendrogram. Second, based on the number of clusters previously defined, the non-hierarchical algorithm K-means was used to establish the members of each cluster. The nearest neighbor algorithm used the Euclidian distance as the distance measure, while the K-means algorithm used the square of the Euclidean distance. Additionally, the K-means algorithm was configured to (i) use random seeds when defining the initial centroids, and (ii) repeat the analysis 100 times and return the most frequent result.

## Results

The results are presented below. First, the development of the survey and the risk inventory are explained. Second, the survey application is described, and finally, the data from the survey responses are analyzed and discussed.

### Survey development: risks and origin

Twenty-eight risks were selected for inclusion in the risk inventory. Table 1 below shows the risks that were identified for each guideline. Five additional risks were added: disputes with insurance companies regarding reimbursements; security – active shooter; financial batch claim emanating from reimbursement reforms; use of social communication networks; and union strikes.

**Table 1:** Risk inventory origin

#	Risks	Guideline and participants					
		COSO	ASHRM	HIROC	NHS	AON	Participants
1	Board governance – poor communication or lack of direction	x	x	x	x		
2	Business Interruption Due to Natural Catastrophe	x	x		x		
3	Clinical batch claim		x		x	x	
4	Conflicts due to organizational hierarchy	x	x	x		x	
5	Cyber security	x					
6	Deficiency in development of technology and innovation	x	x	x		x	
7	Dependence on insurance companies			x			
8	Dispute with insurance companies on reimbursements						x
9	Electronic Health Record (EHR)		x	x			
10	Environment Protection Agency or similar				x		
11	External media communication		x	x		x	
12	Financial batch claim emanating from reimbursement reform						x
13	Fraud committed by a provider	x	x	x	x		
14	Government instability	x	x			x	
15	Loss of accreditation			x	x		
16	Non-compliance with laws and regulations	x	x	x	x		
17	Loss of Occupational Safety and Healthcare Administration (OSHA in USA)		x	x	x		
18	Organizational culture	x	x	x	x	x	
19	Physician wellness		x	x		x	
20	Relation between the School of Medicine or Residency program and hospital			x			
21	Active Shooter						x
22	Sentinel events		x	x	x		
23	Supply chain		x	x			
24	Talent retention	x	x		x	x	
25	Terrorism		x			x	
26	Unethical conduct	x	x	x		x	
27	Union strike						x
28	Use of social communication networks						x

A document that includes risk descriptions, risk scenarios, and risk impacts was developed to constitute the healthcare enterprise risk inventory. One of the objectives of the inventory was for the interviewees to have a common understanding of each risk so that meaningful results and comparisons could be obtained. Another objective of the risk inventory was to educate risk managers and other interested professionals. The complete risk inventory is presented in Additional file 3 through an online link.

One concern that was raised in the interviews with the risk managers related to the lack of a common definition of a defined risk. The ERM guidelines currently in place do not offer sufficiently detailed definitions to allow for proper comparisons. For example, regarding the risk of fraud, stealing money from Medicare is fraud, but taking a photograph of a medical record is also fraud. With no explicit definition, individuals may think of the risk of fraud in different ways. A large organization must create a taxonomy to develop a common understanding of identified risks. The risk inventory created should help guide risk managers and other users from different levels, backgrounds, positions, and locations. In addition, if different organizations use the same inventory, it will be possible to develop risk benchmarks around the business aspects of healthcare.

Another new element that the risk inventory provides is the association of each risk with the dimension that the risk impacts. The dimensions used are the patient, for risks that impact the patient’s care or the patient’s family; financial, for risks that impact the organization’s finances; legal or regulatory, for risks that are associated with lawsuits or regulations; reputation, for risks that can impact the hospital’s image; and social, for risks that can affect the region around the hospital or a large number of people.

Finally, the risks are categorized by group using the ASHRM domains and COSO factors as guidelines. The groups are important for the risk analysis and risk assessment phases. Table 2 below lists the enterprise risk events, their groups, the risk descriptions and the impact dimensions.

**Table 2:** Risk inventory – group and impacts

			Risk impact				
Risk	Risk group	Short description	Patient	Financial	Reputation	Legal	Social
Board governance – poor communication or lack of direction	Financial	Relationship with shareholders and the board of the organization; transparency in the information and results, capacity to prosecute governance. Mergers and Acquisitions. Conflict of Interest		x	x		
Business Interruption Due to Natural Catastrophe	Operational	Occurrence of internal or external events, which make it impossible for an organization to maintain its critical activities. Natural disasters must be allocated to this event. Earthquake or Hurricane.	x	x			x
Clinical batch claim	Clinical	With the increase of technologies and multiples techniques applied to patient to treat diseases, the batch claims have increased in size and frequency. Batch claims are frequently related to poor delivery of clinical service.	x	x	x		x
Conflicts due to organizational hierarchy	People	Responsibilities, leadership and respect among the employees and functions. The relationship between the decision-making process and hierarchy. The medical hierarchy needs to be balanced in favor of teaching, learning and patient safety rather than the exercise of power (WALTON, 2006).	x				
Cyber security	Information Technology	Invasion of an internal or external hacker that causes damage to the information security of the organization or its operational capacity. The use of ransomware is frequently present.	x	x	x	x	x
Deficiency in development of technology and innovation	Clinical	Lack of technologic innovation or development of innovations that do not meet the organization’s needs. It is related organization’s ability to possess, dominate and use technological resources that have an effect on its operations. Effects on the quality of clinical procedures and patient experience, as well as valuation of the institution towards insurers can be perceived.	x	x	x		
Dependence on insurance companies	Financial	Negotiations with one health insurance company that accounts for 30% of the billing. The insurance company wants to reduce reimbursements for many medical tests and procedures.	x	x			
Dispute with insurance companies on reimbursements	Financial	An insurance company disputes the drugs, devices, or procedures used by the providers and hospital. The insurance company denies coverage.	x	x		x	
Electronic Health Record (EHR)	Information Tecnology	Difficulty in obtaining information due to error in communication, loss of processing power or difficulty in operating the Hospital’s system.	x			x	
Environment Protection Agency or similar	Compliance	Government agency comes to investigate and fines the hospital or a department of the hospital.	x	x	x	x	x
External media communication	Information Tecnology	Healthy external marketing and media communication about the hospital and close relations. Organizational information being shared before the formal process and department of the hospital. The information timing can’t be the correct, or the information credibility can cause future problems.	x		x	x	
Financial batch claim emanating from reimbursement reform	Political	Financial risk for healthcare organizations associated with bundled services or healthcare outcomes.		x	x		x
Fraud committed by a provider	Financial	Insurance plan fraud committed by a doctor or a group of doctors through prescriptions. In addition, important medicines or equipment stolen from the hospital can also be considered like a fraud.	x	x	x	x	x
Government instability	Political	Reduction in the country’s healthcare budget	x	x			x
Loss of accreditation	Compliance	Loss of an important certification or accreditation.	x	x	x	x	
Non-compliance with laws and regulations	Compliance	A clinical trial is taking place without the proper Institutional Review Board (IRB) approval. Patients die while part of the research.	x	x	x	x	x
Loss of Occupational Safety and Healthcare Administration (OSHA in USA)	Compliance	The effect that working laws represent in how employees are being contracted. Any change in the formal orientations represent an effect for the hospital management.	x	x		x	
Organizational culture	People	The healthcare organization needs to be able to share and implement its culture among all the employees. New and old employees need to work conducted by the same values and principles independently of their own religion or origins.	x				
Physician wellness	People	50% rate of burnout amongst physicians discovered after taking a physician wellness survey that measures burnout and professional fulfillment.	x	x		x	
Relation between the School of Medicine or Residency program and hospital	Clinical	Interface between the SoM and the health service that may lead to interference of the university model to the business or, on the other hand, value the institution due to the teaching quality.	x		x		x
Active Shooter	Operational	Assault and active shooter threats to patients, families and hospital employees.	x		x	x	x
Sentinel events	Clinical	Sentinel events, near miss events, incidents or medical error that can cause lawsuit.	x	x	x	x	
Supply chain	Operational	Materials and equipment control and management. Political problems with countries that supply resources for hospitals.	x	x			x
Talent retention	People	Loss of a team of providers that are specialized in certain types of procedures. It can happen in function of bad recruitment processes, or bad human resources management.	x	x	x		x
Terrorism	Political	Terrorism attack close to the hospital.	x	x	x	x	x
Unethical conduct	Operational	Problems related with unethical employee conduct whether or not involving patients. Personal information, images or objects can be used without the approval of patient. Internal problems between employees can result in organization impact.	x	x	x	x	x
Union strike	Political	Union strikes among different classes of employees that can affect the hospital capacity to be operated.	x	x	x	x	
Use of social communication networks	Information Tecnology	Problems with confidential information being communicated through social media. A VIP: executive, actor, etc. Information is released on Facebook, what’s app or other.	x	x	x		x
		Total/impact	26	22	18	15	15

### Survey application

**Fig. 2:**
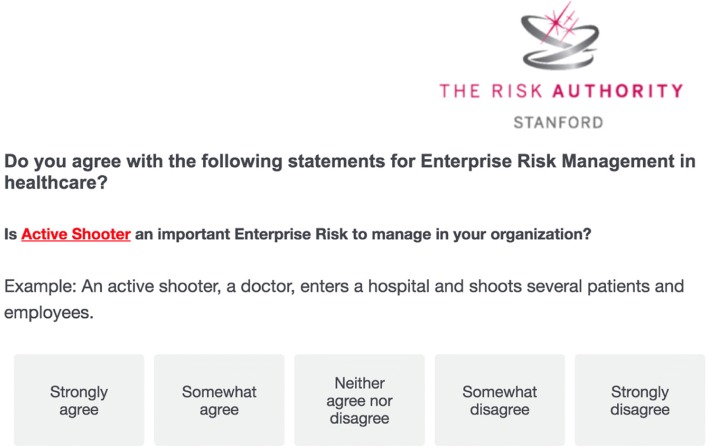
Risk inventory survey example

After the risk inventory was completed, the survey was developed. For each risk, the participants were asked if they strongly disagreed, somewhat disagreed, neither disagreed nor agreed, somewhat agreed or strongly agreed that the risk is an important enterprise risk in the healthcare industry. The survey was anonymous. To create strata and analyze the responses, additional questions were asked to determine the credentials of the participants and the type of institution in which they work. The questions were used to determine the participants’ position, years working in that position, number of employees in the company, and whether a school of medicine was present. This information was used to develop the sample strata. Figure 2 presents an example of the risk questions on the platform.

A total of 69 risk professionals started the survey, and 53 completed surveys were obtained during the period of study. This sample did not reach our 5% confidence interval target; however, it is still under the 10% confidence interval (required sample size of 44 participants).

### Data analysis

The survey data were exported to a CSV file, and the software SPSS was used to conduct the analysis. A total of 28 participants believed that their organization had a very or moderately effective ERM program. Thirty-eight participants worked in non-for-profit organizations, and 35 were from organizations with a school of medicine or a residency program. Twenty-seven participants were chief risk officers or executive professionals, and 26 were clinical risk managers. A total of 19 participants had worked fewer than 7 years in risk management, and 34 had more than 7 years’ experience working in risk management. Finally, 26 participants worked in organizations with fewer than 1000 employees, and 27 worked in organizations with more than 1000 employees.

**Fig. 3:**
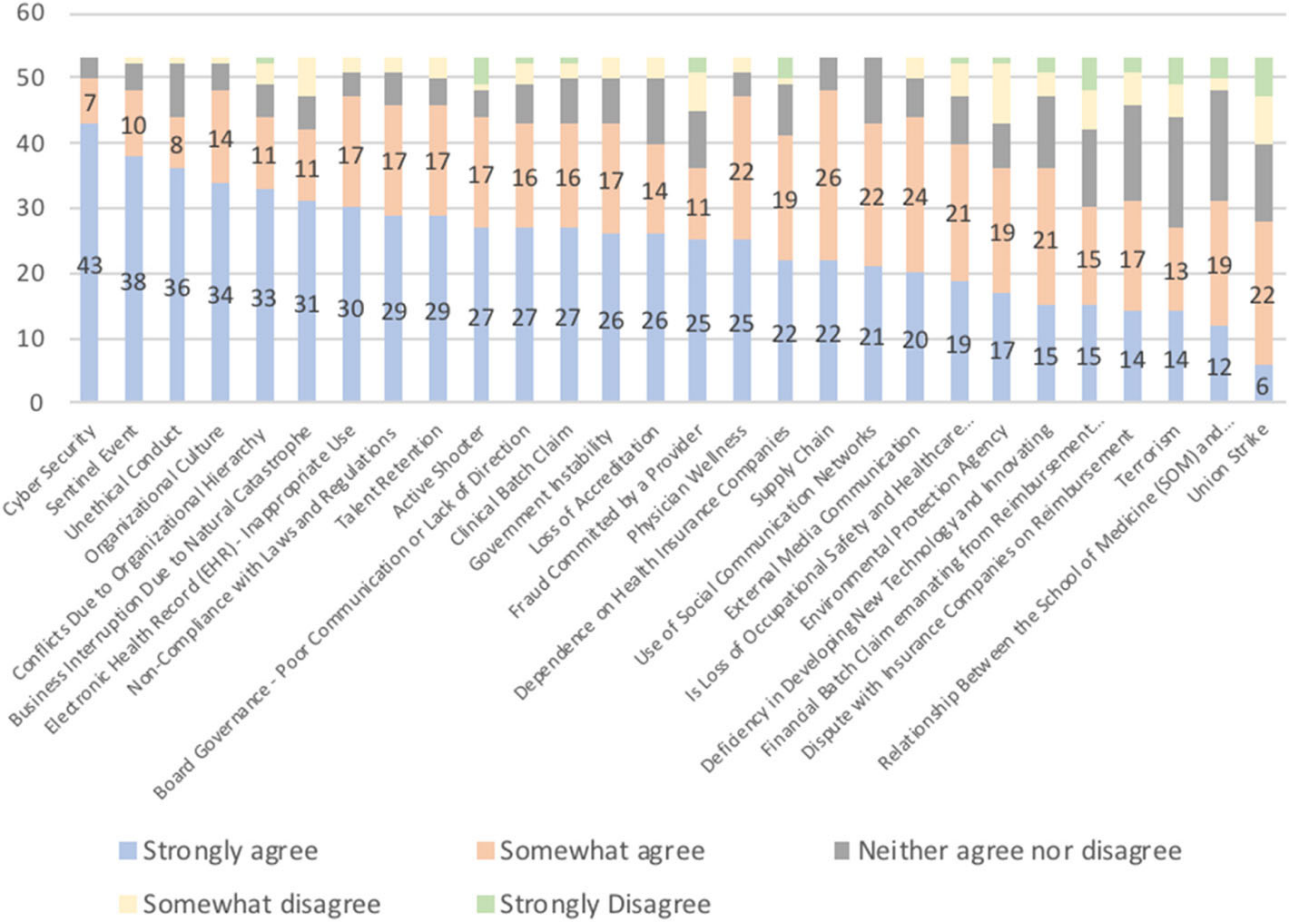
Risk ranking according to the 53 participants

The first analysis aimed to develop a ranking of the 28 risks. Figure 3 shows the risk ranking ordered by the perceived level of risk importance. The y-axis refers to the frequency with which each risk was identified, and each color bar shows one of the alternative choices.

Cyber security was ranked first, which highlights the importance that risk managers have placed on cyber issues. The second highest ranked risk was “sentinel event”. This result was expected, given the number of international regulations and rules to monitor and control sentinel events.

The sentinel events, unethical conduct, organizational culture, and conflicts due to organizational culture risks demonstrate the importance of employee management in the healthcare industry. These risks are associated with an organization’s ability to manage human capital in alignment with respect for the values, rules and objectives established by organizational leaders.

**Fig. 4:**
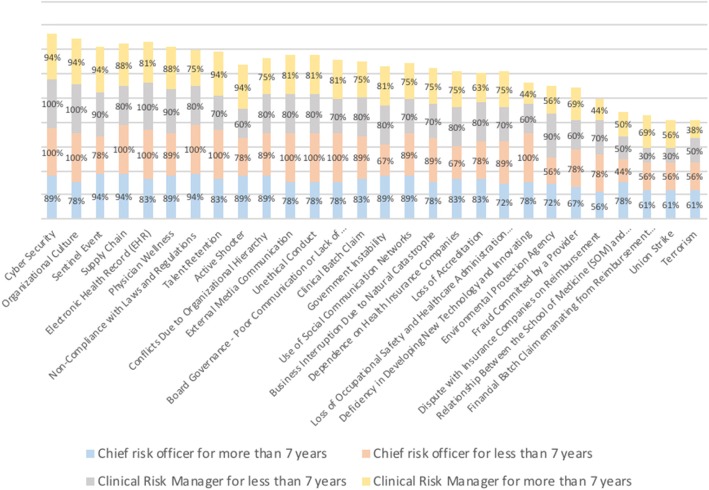
Type of risk management and time working in risk management

The second analysis (Fig. 4) shows the differences in the answers for the four groups representing the strata detailed in the methods section. The y-axis represents the percentage of each group that agree that the risk is an important enterprise risk: i) chief risk officers with more than 7 years working in risk management, 18 participants; ii) chief risk officers with fewer than 7 years working in risk management, 9 participants; iii) clinical risk managers with more than 7 years working in risk management, 10 participants; and iv) clinical risk managers with fewer than 7 years working in risk management, 16 participants.

Figure 4 shows that chief risk officers tend to agree more than clinical risk managers regarding the risks that they consider to be important. The average percentage in which chief risk officers answered that they strongly agreed or somewhat agreed on the importance of each risk was 83% (the blue and orange bars in Fig. 4). In contrast, the percentage for clinical risk managers was 73% (the gray and yellow bars in Fig. 4). When considering the type of risk management, the difference in the average percentage with regard to years of experience is small: 76% for more than 7 years and 78% for fewer than 7 years working in risk management.

**Fig. 5:**
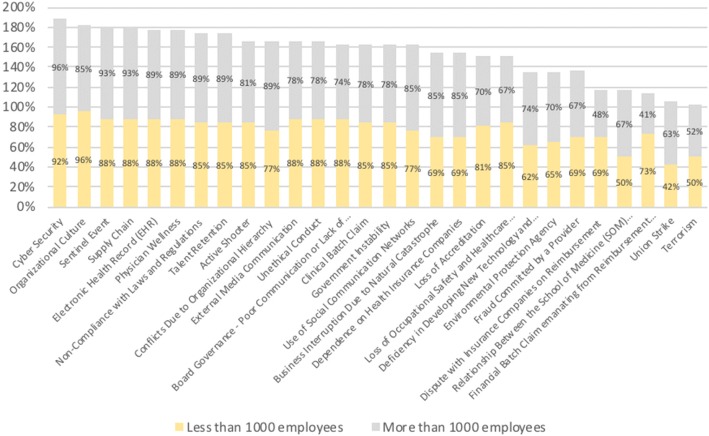
Differences between participants from hospitals with fewer and more than 1000 employees

The results shown in Fig. 5, the third analysis, are similar to those in the second analysis. The y-axis represents the percentage of participants from each group that agree that the risk is important. The figure shows that the size of a healthcare organization has no impact on risk professionals’ perception of risks: the average percentage in which participants from organizations with fewer than 1000 employees (27 participants) answered that they strongly agreed or somewhat agreed on the importance of each risk was 77% (yellow bar). On the other hand, the same percentage for the group from companies with more than 1000 (26 participants) employees was 76% (gray bar).

**Fig. 6:**
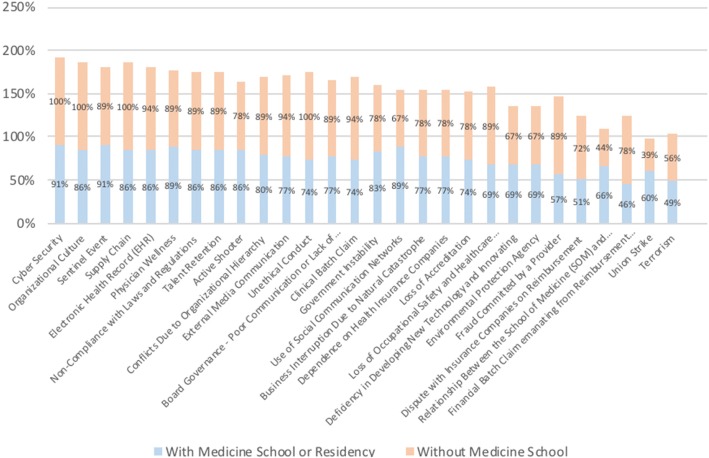
Differences between participants working in organizations with and without a school of medicine

With regard to the presence of a school of medicine, it is possible to identify small differences in the perceptions of risks between the two groups. In general, the managers from organizations without a school of medicine or residency program (18 participants) tend to agree slightly more about the importance of each risk than those with a school of medicine or residency program (35 participants), on average 6% more. However, for the following risks, the opposite is true, i.e., those who work in an organization with a school of medicine or residency program agree more about the importance of the following risks: security – active shooter, government instability, use of social media networks, deficiency in developing new technology and innovation, relation between the school of medicine and hospital and union strikes. Figure 6 shows the results, with the y-axis indicating the percentage of participants who agree about the importance of the risk from each group.

The cluster analysis defined four different groups, as shown in Table 3.

**Table 3:** Number of cases per cluster

Number of cases in each cluster		
Cluster	1	15.000
	2	34.000
	3	1.000
	4	3.000
Valid		53.000
Missing		.000

The K-means algorithm was used to establish which participants were included in each of the four clusters. Table 4 shows the results.

**Table 4:** Cluster membership

Case number	Cluster	Distance
17	4	7.659
21	4	9.309
23	4	7.394
6	3	.000
2	2	8.886
4	2	6.735
5	2	9.196
7	2	6.615
8	2	5.874
9	2	6.131
10	2	6.537
11	2	6.927
12	2	8.148
13	2	8.315
14	2	8.841
15	2	7.964
16	2	6.633
18	2	5.666
19	2	7.694
20	2	12.066
24	2	8.969
26	2	7.694
27	2	7.179
30	2	11.298
32	2	7.398
34	2	6.769
39	2	11.399
41	2	5.686
42	2	9.645
43	2	5.366
44	2	7.195
46	2	10.550
47	2	6.422
50	2	5.461
52	2	6.633
53	2	6.242
37	2	8.783
38	2	9.007
1	1	10.223
3	1	11.056
22	1	13.069
25	1	8.844
28	1	9.005
29	1	10.025
31	1	9.911
33	1	7.960
35	1	12.624
36	1	10.444
40	1	9.997
45	1	9.036
48	1	7.046
49	1	7.779
51	1	11.854

Clusters 1 and 2 include 92% of the sample. The remaining 8% is divided among cluster 3 (one member) and cluster 4 (three members). Case 6 is a single member of cluster 3, which can be explained by the fact that the individual is a health insurance broker focused on clinical insurance. The members of cluster 4 are clinical risk managers working in not-for-profit companies with established ERM processes.

Subsequently, ANOVA was used to identify which questions had statistical significance to the establishment of participant group membership. Table 5 presents the results.

**Table 5:** Analysis of variance of cluster members

Questions	Cluster mean square	df	Error mean square	df	F	sig.
Clinical Batch Claim	27.262	3	2.234	49	12.204	.000
Conflicts Due To Organizational Hierarchy	28.863	3	2.493	49	11.577	.000
Dependence on health insurance	47.235	3	2.074	49	22.777	.000
Dispute with insurance companies in reimbursement	33.268	3	2.987	49	11.136	.000
Environmental protection agency	32.966	3	3.428	49	9.617	.000
External media communication	16.074	3	2.019	49	7.960	.000
Fraud commited by a provider	67.311	3	2.077	49	32.411	.000
Non-compliance with laws and regulations	20.580	3	1.552	49	13.257	.000
Loss of occupational safety and healthcare administration (OSHA in USA)	23.920	3	3.024	49	7.911	.000
Physician wellness	16.601	3	1.559	49	10.650	.000
Sentinel event	16.908	3	1.143	49	14.797	.000
Board governance-Poor communication or lack of direction	19.512	3	2.992	49	6.522	.001
Active shooter	23.871	3	4.210	49	5.670	.002
Financial batch claim emanating from Reimbursement reform	30.082	3	5.108	49	5.889	.002
Cyber security	5.239	3	.970	49	5.401	.003
Unethical conduct	11.637	3	2.165	49	5.376	.003
Supply chain	6.196	3	1.380	49	4.491	.007
Union strike	18.470	3	4.808	49	3.841	.015
Business Interruption due to natural catastrophe	13.759	3	3.754	49	3.666	.018
Relationship between the school of medicine (SOM) and Hospital	13.718	3	3.886	49	3.531	.021
Electronic Health Record (EHR)	7.338	3	2.234	49	3.285	.028
Terrorism	14.222	3	5.269	49	2.699	.056
Organizational Culture	4.998	3	1.915	49	2.610	.062
Loss of Accreditation	8.363	3	3.252	49	2.571	.065
Government Instability	7.226	3	2.971	49	2.432	.076
Deficiency in developing new technology and innovating	8.506	3	4.223	49	2.014	.124
Talent retention	5.491	3	2.781	49	1.974	.130
Use of social communication networks	2.744	3	2.176	49	1.261	.298

Table 5 shows that only 13% (7) of the questions were not significant to the identification of cluster members. This result indicates that the risks integrated in the enterprise risk inventory captured each risk’s perceived importance during the survey application because the analysis indicates that the majority of the risks were significant to the cluster formation.

**Fig. 7:**
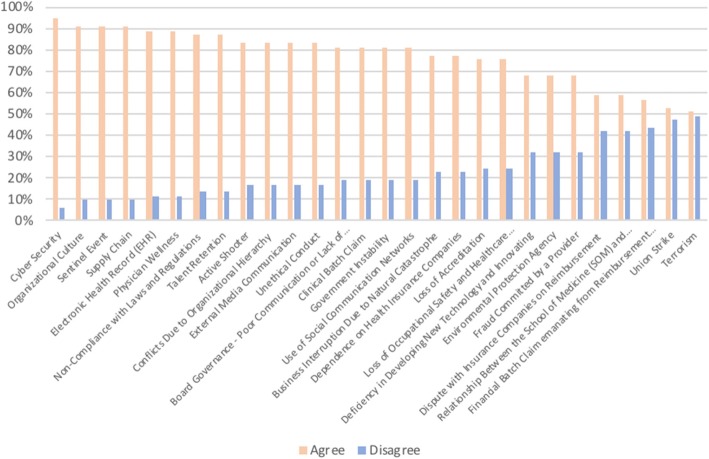
Agree x disagree - risk inventory confirmation

Furthermore, the final analysis shows that all 28 risks were confirmed through the survey. More than 50% of the managers somewhat agreed or strongly agreed that all the risks are important enterprise risks in the healthcare industry, and this percentage is higher than 70% for 20 risks (all risks above and including loss of occupational safety and healthcare administration in Fig. 7). This represents an important advance in healthcare risk research and for practical application. Figure 7 shows the results, with the y-axis indicating the percentage of participants who agreed or disagreed that the risks are important.

The final analysis was performed to examine the free text written by the participants in response to an additional comments question. One item was mentioned by many participants: the importance of managing the impact of a hospital’s external image. The risk inventory does not include reputation as a risk but rather as an impact. However, two additional risks were reported. First, the participants mentioned investments in outpatient care and their connection to the hospital’s capability to deliver a positive patient experience. The second risk mentioned was the growth in healthcare technology that has enabled home healthcare throughout the world. This risk impacts the support patients receive from the hospital after hospitalization.

## Discussion

ERM is applied across the board and is subject to the strategic positioning of organizations, which have the autonomy to manage processes and provide informational support when making strategic decisions [12]. When conducting ERM program, it is important that employees with different expertise and from different positions work together to incorporate the specific characteristics of the market in which the program will be implemented [10, 12, 22]. Therefore, it is particularly important for individuals with diverse expertise and experience to have a common understanding and specific definitions of risk events [11]. The results of this study show that having a personal background in risk management (clinical or enterprise) was a characteristic that showed a small difference in the perceived importance of the risks from the proposed risk inventory. These results highlight the necessity for clinical risk managers to work closely with chief risk officers to create a risk culture across the entire organization [9, 12, 23]. The length of time working in risk management and the number of employees in an organization do not show substantial differences with regard to the answers in Figs. 4 and 5. The cluster analysis also confirmed these results, as participants’ background had no influence on cluster membership. Both types of backgrounds were found in participants in all 4 clusters.

With regard to the small difference in how risk managers from organizations with and without a school of medicine agree with the risk results, an argument made during the first phase of the interviews (15 managers) deserves attention. A possible explanation is that organizations associated with schools of medicine are more exposed to students posting on social media than organizations that have formal contracts with employees [24]. Additionally, schools of medicine connect organizations to government funding, which can lead to instability. Hospitals associated with schools of medicine and residency programs also must contribute to research and innovation capability [25].

When ASHRM started to include ERM in its own principles in 2011, one objective was to connect ERM concepts from other industries to the traditional risk management concepts present in healthcare organizations [26]. The developed risk inventory innovates in the risk identification phase of ERM by highlighting ways that enterprise risks affect patient care. Of the 28 risks identified, 26 can impact patient care or the patient’s family. ERM teams in healthcare organizations need to develop transparent processes that include the clinical impact of risks, irrespective of whether the initial risk event was clinical. This approach would help make patient care and the patient experience the focus to guide the strategic decision-making process.

With regard to the other characteristics explored among the study participants, it is possible to assume a near consensus regarding risk perceptions independent of the type of risk management performed or the length of time working in risk management, as demonstrated by the cluster analysis. Although we were not able to identify participant characteristics that lead to membership in clusters 1 and 2, the presence of clinical risk managers, chief risk officers, and employees with different levels of experience working in risk management led us to conclude that the length of time working in risk management as well as the participants’ background had no influence on cluster membership. Therefore, as the previous descriptive statistics analysis suggests, it is possible to assume that risk perceptions are not directly associated with the length of time working in risk management and the type of risk management performed (clinical or enterprise). This result can be explained by the fact that a risk manager in a healthcare organization is involved in many areas: accounting, actuarial sciences, the healthcare business, information technology, and people management, among others [9, 12]. Thus, the organizational structure does not greatly affect the way that risk managers think about risk. Some participants reported that it is individuals’ responsibility to stay current on the innovations in risk management and to be completely engaged in the cause.

Cyberattack-related risk was identified as the number one enterprise risk for healthcare organizations, and this result is supported by the attention and investment allocated to combatting hackers. The last report developed by AON suggests that healthcare organizations are increasingly purchasing data breach coverage to protect their sensitive patient information [27]. This is mainly driven by the HIPAA legislation, which outlines data privacy and security provisions for safeguarding medical information and that now holds organizations responsible in the event of a breach [27].

The year 2017 will be remembered for the large number of cyberattacks targeting healthcare organizations. Hackers accessed hospital databases throughout the world, interrupting operations and stealing data from millions of patients and thousands of companies. The National Health Service in England and Scotland announced in May 2017 that it would spend €60,000,000.00 per year on the NHS’ cyber system to improve its security [28]. By August 2017, the healthcare sector reported 233 breach incidents to the US Department of Health and Human Services in which more than 3.16 million patient records were breached [29]. These events align with the results found in this research and justify the investments in research and the dollars spent to improve information systems to keep hospital data safe.

Patient data security in hospitals that operate with high levels of technology is fundamental to delivering high quality and safe care to patients. The identification of cyberattacks at the top of the risk ranking reflects the importance that healthcare risk managers are placing on this risk by allocating time and other resources.

Sentinel events are a specific characteristic of healthcare organizations, which have been encouraged by international institutions such as JCI to reduce sentinel events through safety and quality practices [30, 31]. Human errors represented a starting point for advances in the clinical risk management literature since the publication of To Err is Human [32] and “Crossing the Quality Chasm” by the Institute of Medicine [33]. These publications suggest that between 3.7–16.7% patients suffer an adverse event, and it is estimated that a half of these events could be prevented through better risk management practices. These events and the attention paid to this issue by international institutions were highlighted during this research, as the participants confirmed the importance of all risks associated with employee management and human relations in healthcare organizations.

Addressing clinical teams’ emotional exhaustion is essential to ensuring a high level of patient safety [34]. Indeed, Wallace et al. [35] concluded that physician wellness might not only benefit the individual physician, it could also be vital to the delivery of high-quality healthcare. The authors suggest that physician wellness may be an organizational indicator of quality [35]. The sequence of risks noted as important in the survey, including unethical conduct, the organizational culture, and conflicts due to the organizational culture are associated human capital management. The fact that healthcare organizations are sustained by human capital is clearly an important issue for risk management [25]. According to some of the interviewed managers, this highlights the necessity of having a well-described risk inventory and a defined risk management process to minimize interpersonal conflicts based on the existence of a document that establishes rules for professionals [19]. The hierarchy among employees in a healthcare organization and professionals’ dependency on such employees deserve attention when implementing a proactive and strategic risk management process because only by engaging all professionals in ERM can a risk culture be created and a safer environment achieved [36–39].

## Conclusion

The results provide important progress for the strategic healthcare management process and ERM programs. In addition to defining specific risk scenarios, the enterprise risk inventory presented in this research can be used to educate professionals, guide the risk identification phase in future ERM programs, and thereby contribute to the development of a risk culture.

Establishing cyberattacks and the risks associated with human capital management (organizational culture, use of electronic medical records and physician wellness) at the top of the risk ranking is an important contribution of this research. Cyber security is at the top of the risk list for most industries, including healthcare. Employee wellness is also a theme that has been growing in importance in many industries. There are now opportunities to investigate and develop solutions to manage and assess those risks for healthcare organizations.

The results also demonstrate that the qualitative characteristics of risk managers from large organizations, the length of time working in risk management, and the presence of a school of medicine do not alter the perceived importance of the risks. Clinical risk managers and chief risk officers have small differences of opinion on the risks, but not enough to group them in the same cluster. This finding enables us to conclude that the personal background of each employee is a more important factor than the organization’s structure or the employee’s own risk perception capability.

For future research, the authors suggest evaluating the benefits of using the risk inventory at the beginning of the risk identification phase, that is, during the baseline phase of the E^2^RMhealthcare. To demonstrate the value of the risk inventory, a comparative study that explores the ability to disseminate an ERM program in an organization should be conducted.

## Additional files

Additional file 1: Interviewees description. (XLSX 35 kb)

Additional file 2: The enterprise risk inventory survey. (DOCX 29 kb)

Additional file 3: The enterprise risk inventory. (DOCX 32 kb)

## Abbreviations

ASHRM: American society for healthcare risk management
COSO: Committee of sponsoring organizations of the treadway commission
ERM: Enterprise risk management
HIPAA: Health insurance portability and accountability act
HIROC: Healthcare insurance reciprocal of Canada
ISO: International organization for standardization
JCI: Joint commission of international standards for hospitals
NHS: The national health service in England
USA: United States of America
