# Supplementary material for: Development of an enterprise risk inventory for healthcare
Source: BMC Health Serv Res. 2018 Jul 24;18:578. doi: 10.1186/s12913-018-3400-7 (PMC6057062; doi:10.1186/s12913-018-3400-7)
Supplement: Supplementary file 3 — The enterprise risk inventory. (DOCX 32 kb) [file 12913_2018_3400_MOESM3_ESM.docx]

<https://drive.google.com/drive/folders/1Qsk2zdGVKTZuOqgR1lYMvYXMbihitLYe?usp=sharing>
